# Predicting protective gene biomarker of acute coronary syndrome by the circRNA-associated competitive endogenous RNA regulatory network

**DOI:** 10.3389/fgene.2022.1030510

**Published:** 2022-10-19

**Authors:** Hengliang Zhang, Daphne Merkus, Pei Zhang, Huifeng Zhang, Yanyu Wang, Laijing Du, Lakshme Kottu

**Affiliations:** ^1^ The First Affiliated Hospital, and College of Clinical Medicine of Henan University of Science and Technology, Luoyang, China; ^2^ Walter-Brendel-Centre of Experimental Medicine, University Hospital, Ludwig-Maximilians-University München, Munich, Germany; ^3^ Department of Experimental Cardiology, Erasmus University Medical Center, Rotterdam, Netherlands

**Keywords:** acute coronary syndrome, circular RNA, competitive endogenous RNA, protective gene biomarkers, immune cells, XPNPEP1 gene

## Abstract

**Background:** The mortality and disability rates of acute coronary syndrome (ACS) are quite high. Circular RNA (circRNA) is a competitive endogenous RNA (ceRNA) that plays an important role in the pathophysiology of ACS. Our goal is to screen circRNA-associated ceRNA networks for biomarker genes that are conducive to the diagnosis or exclusion of ACS, and better understand the pathology of the disease through the analysis of immune cells.

**Materials and methods:** RNA expression profiles for circRNAs (GSE197137), miRNAs (GSE31568), and mRNAs (GSE95368) were obtained from the GEO database, and differentially expressed RNAs (DEcircRNAs, DEmiRNAs, and DEmRNAs) were identified. The circRNA-miRNA and miRNA-mRNA regulatory links were retrieved from the CircInteractome database and TargetScan databases, respectively. As a final step, a regulatory network has been designed for ceRNA. On the basis of the ceRNA network, hub mRNAs were verified by quantitative RT-PCR. Hub genes were validated using a third independent mRNA database GSE60993, and ROC curves were used to evaluate their diagnostic values. The correlation between hub genes and immune cells associated with ACS was then analyzed using single sample gene set enrichment analysis (ssGSEA).

**Results:** A total of 17 DEcircRNAs, 229 DEmiRNAs, and 27 DEmRNAs were found, as well as 52 circRNA-miRNA pairings and 10 miRNA-mRNA pairings predicted. The ceRNA regulatory network (circRNA-miRNA-mRNA) was constructed, which included 2 circRNA (hsa_circ_0082319 and hsa_circ_0005654), 4 miRNA (hsa-miR-583, hsa-miR-661, hsa-miR-671-5p, hsa-miR-578), and 5 mRNA (*XPNPEP1, UCHL1, DBNL, GPC6,* and *RAD51*). The qRT-PCR analysis result showed that the *XPNPEP1, UCHL1, GPC6* and *RAD51* genes had a significantly decreased expression in ACS patients. Based on ROC curve analysis, we found that *XPNPEP1* has important significance in preventing ACS occurrence and excluding ACS diagnosis. ACS immune infiltration analysis revealed significant correlations between the other 3 hub genes (UCHL1, GPC6, RAD51) and the immune cells (Eosinophils, T folliculars, Type 2 T helper cells, and Imumature dendritic cells).

**Conclusion:** Our study constructed a circRNA-related ceRNA network in ACS. The *XPNPEP1* gene could be a protective gene biomarker for ACS. The *UCHL1, GPC6 and RAD51* genes were significantly correlated with immune cells in ACS.

## Introduction

One of the most serious, urgent, and lethal disorders in the clinic is acute coronary syndrome (ACS), which includes unstable angina pectoris (UA) and acute myocardial infarction (AMI). In high-income nations, the incidence of ACS is 200–250 cases per 100,000 person-years, and ACS is responsible for one-third of all mortality (Sanchis-Gomar et al., 2016; Ibanez et al., 2018; Collet et al., 2021). The exact pathogenic mechanism of ACS has not yet been extensively investigated. An early diagnosis, early intervention, and early prevention are essential tools in reducing ACS’s harm and improving its prognosis. Researchers are investigating ACS′ pathogenic process at the protein, molecular, and gene levels simultaneously. (Sposito, 2022; van den Broek and Ten Berg, 2022). Finding the genes that promote or protect ACS is critical since it can fundamentally prevent disease and give a theoretical foundation for the development of targeted medicine and precision therapy.

As a competitive endogenous RNA (ceRNA), circular RNA (circRNA) plays a significant role in the pathogenesis of ACS. CircRNA affects gene transcription and regulation by interacting with miRNA, mRNA, or protein *via* the ceRNA mechanism (Hansen et al., 2013; Zhao et al., 2018). In recent years, more evidence has emerged demonstrating that circRNA expression dysregulation plays a critical role in the ceRNA regulatory network and is a fundamental etiology of atherosclerotic diseases such as acute cerebral infarction and acute coronary syndrome (Zhao et al., 2020). In order to further clarify how circRNA affects the occurrence and development of ACS, we set up a circRNA-associated ceRNA network to investigate the pathophysiology of ACS at the molecular level. Through the analysis of the hub genes in the ceRNA network, we will try to screen the biomarker genes that are conducive to the diagnosis or exclusion of ACS, and explore its pathological mechanism through immune cell analysis.

## Materials and methods

### Data collection

The Gene Expression Omnibus database (GEO, https://www.ncbi.nlm.nih.gov/geo/) plays an important role in many fields, including comparative genomic analysis, proteomics, non-coding RNA, single nucleotide polymorphism genome and gene methylation status analysis (Sayers et al., 2022). A search was conducted from the GEO dataset for microarrays that met the following requirements: using “acute coronary syndrome” OR “unstable angina” OR “acute myocardial infarction” AND “circRNA”.

**FIGURE 1:**
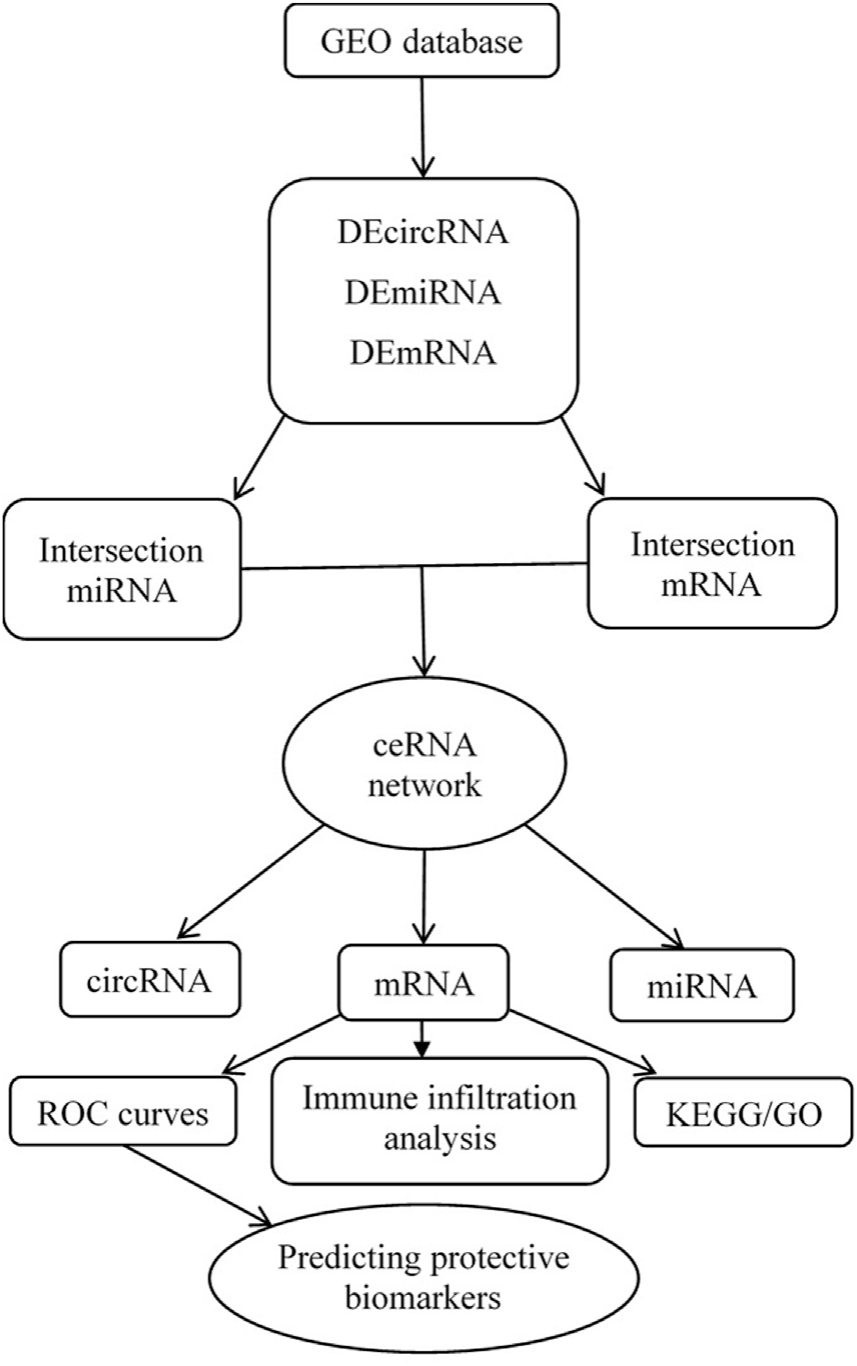
Flow chart for the construction of the circRNA-associated ceRNA regulatory network and analysis of the hub genes.

Three datasets were downloaded from the GEO database after a selection process. One circRNA expression profiling dataset [GSE197137 (GPL21825 platform)], consisted of 3 ACS patients and 6 subjects without ACS as controls. One miRNA expression profiling dataset [GSE31568 (GPL9040 platform)] included 20 ACS patients and 70 healthy controls, and one mRNA expression profiling dataset [GSE95368 (GPL23119 platform)] included 12 ACS patients and 6 healthy controls. Our research process is shown in Figure 1.

### Data processing

The genes used for constructing the ceRNA network must be differentially expressed (DE) in ACS patient samples compared to non-ACS subject samples. Perl software version 5.30.0.1 was used to convert the probe matrix into an RNA matrix. DEcircRNA, DEmiRNA, and DEmRNA were obtained using the “limma” package in R version 4.0.3. The following screening criteria were considered statistically significant: | log_2_ fold chang | > 2 and adjusted *p*-value < 0.05. The pheatmap package, ggpubr package and reshape2 package in R are used for the visual analysis of differentially expressed RNA.

### Construction of the competitive endogenous RNA network

According to the theory that circRNAs act as miRNA sponges in mammalian cells (Salmena et al., 2011), we constructed a circRNA-miRNA-mRNA regulatory network. The CircInteractome database (https://circinteractome.nia.nih.gov/index.html) was used to predict miRNA binding sites (MREs) (Dudekula et al., 2016). The circRNAs’ chromosomal position, as well as the chromosomal location and length of the RNA necessary for the investigation, were all provided by CircBase. TargetScan databases were used to anticipate interactions between intersection miRNAs and target mRNAs (Xiong et al., 2018). Finally, the data for the regulatory network (circRNA-miRNA-mRNA) was processed through Perl software, and then a visual ceRNA regulatory network was established by using Cytoscape 3.8.0 software.

### KEGG and GO pathway enrichment analysis

GO analysis (http://geneontology.org/) covers three areas, cellular components (CC), molecular functions (MF) and biological processes (BP). Each category explains the biological function of genes at different levels (Zhang et al., 2019). The Kyoto Encyclopedia of Genes and Genomes (KEGG) database (https://www.kegg.jp/) is a popular public resource for learning about the degree of enrichment of differential genes in pathway terms (Kanehisa and Goto, 2000). We performed GO function annotation and KEGG pathway analysis to further investigate the pathway and mechanism of DEmRNA in the ceRNA network affecting ACS by using the “cluster profiler” package in the R software.

### Quantitative RT-PCR verification

We obtained peripheral blood from the patients with ACS who visited the emergency department within 12 h of the onset of chest pain. Total RNA was isolated from blood using Trizol and cDNA was synthesized using reverse transcription kits (Takara, Beijing, China) according to the manufacturer’s instructions. Quantitative reverse transcription PCR was performed using SYBR Green Mix on an ABI7900HI (Thermo Fisher Scientific). The program was set to be a two-step method, 95°C for 5 s, 60°C for 30 s, and 40 cycles. The gene expression results were analyzed using the 2^^−ΔΔCT^ method, and GAPDH was used as an endogenous control for mRNA expression. The primer sequence information of the qRT-PCR experiment is shown in Supplementary Table S1.

### Predicting the protective value of characteristic biomarkers in acute coronary syndrome

In order to test the protective value of identified biomarkers, receiver operating characteristic (ROC) curves were generated using the mRNA expression data from the GSE95368 and another independent mRNA database GSE60993. The predicting protective values of the identified hub genes were evaluated using the area under the ROC curve (AUC), which was between 0.5 and 1. The closer the AUC is to 1, the better the predictability of the protective effect. We obtained patient follow-up data from the GSE95368 dataset and used logistic regression analysis to investigate the relationship between the hub gene and major adverse cardiovascular events (MACE).

### Expression of XPNPEP1 in cardiomycytes by immunofluorescence staining

We obtained human induced pluripotent stem cells (iPSC) from skin fibroblasts of healthy human donor and ACS patients. We referred to Chen’s scheme to differentiate the hiPSC to cardiomyocytes (Chen et al., 2011). Cardiomyocytes derived from human induced pluripotent stem cells (hiPSC-CM) were fixed with 4% paraformaldehyde for 1 h. After permeabilization (1% Triton X-100, 1 h), staining was performed with the primary antibody (anti-rabbit XPNPEP1 antibody, 1:50, Abcam), appropriate secondary antibody (goat anti-rabbit IgG Alexa Fluor 594, 1:100, Invitrogen) and DAPI (Invitrogen). The stained cytoskeleton structure was observed under a laser confocal microscope (LSM800; Carl Zeiss Meditec, Jena, Germany).

### Correlation analysis between acute coronary syndrome hub genes and immune infiltrating cells

The single sample gene set enrichment analysis (ssGSEA) method was then used to evaluate the abundance of 23 immune cells in ACS to further investigate the correlation. Then we analyzed the correlation between hub genes and ACS related immune cells. The heat map and the vioplpt were constructed by the “ggplot2” package and the “ggpubr” package to visualize the features.

## Results

### Extraction of DEcircRNA, DEmiRNA, and DEmRNA

**FIGURE 2:**
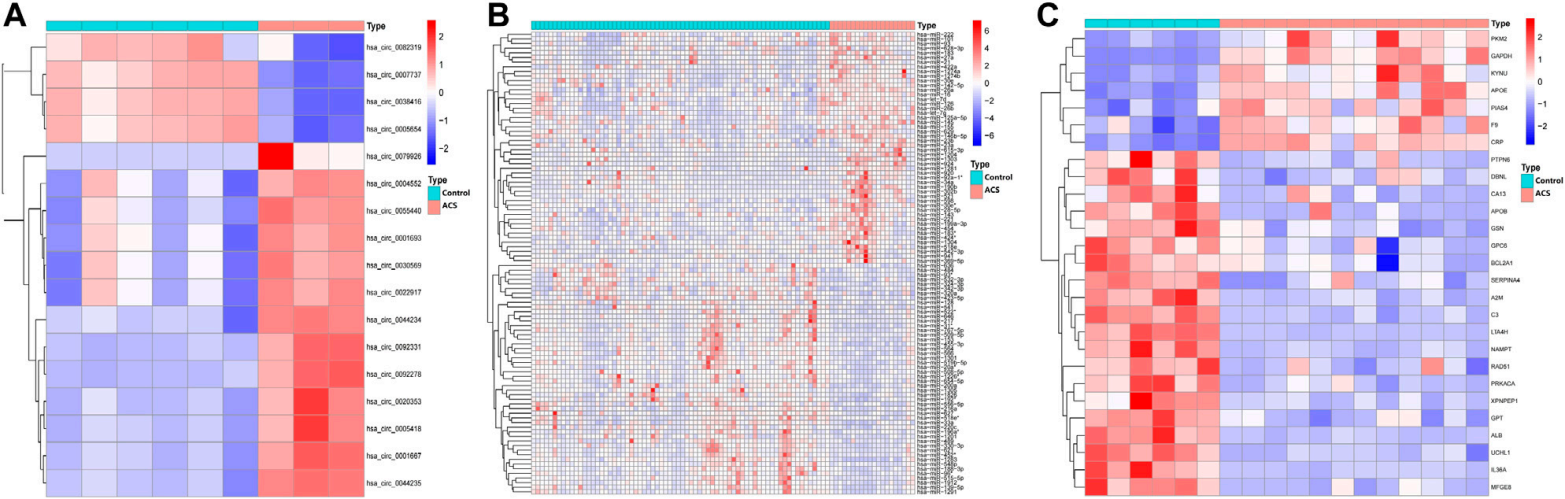
Heatmap of differentially expressed circRNA, miRNA, and mRNA. Red represents upregulated expression, and blue means downregulated expression. **(A)** 17 differentially expressed circRNAs in the GSE197137 dataset. **(B)** Top 50 of 229 differentially expressed miRNAs in the GSE31568 dataset. **(C)** 27 differentially expressed mRNAs in the GSE95368 dataset (|log_2_ fold change|>2, adjusted *p*-value<0.05).

Human-derived RNA datasets (GSE197137, GSE31568, and GSE95368) containing patients with ACS and subjects without ACS were selected. The differential expression of the data set was analysed with the “limma” package. In total, 17 DEcircRNAs were obtained, of which 4 were downregulated and 13 were upregulated. Of the 229 DEmiRNAs, 103 were downregulated and 126 were upregulated. There were 27 DEmRNAs, 20 were downregulated and 7 were upregulated. As shown in Figure 2, we chose the top 50 downregulated and upregulated DERNAs for heat map analysis.

### Identification of target miRNA and mRNA of circular RNA

**FIGURE 3:**
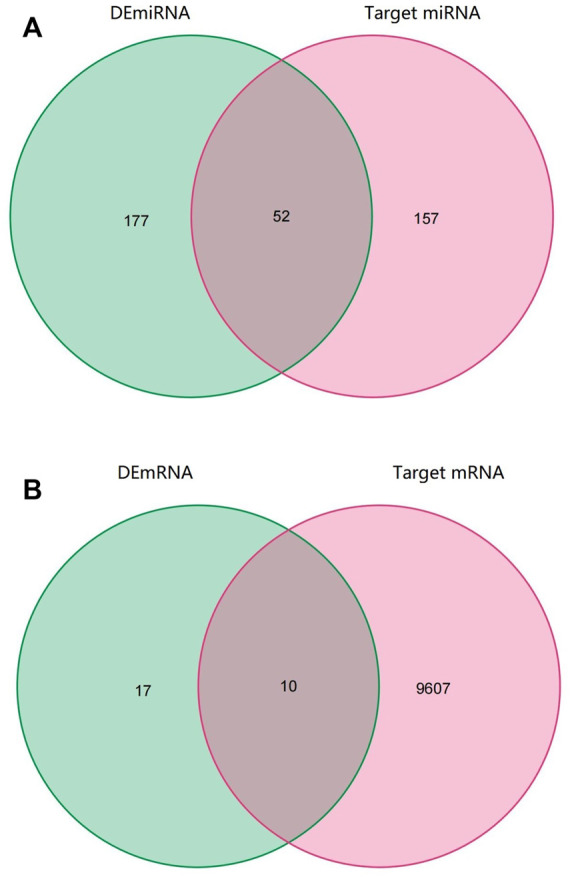
Venn diagram of RNAs involved in the ceRNA network. **(A)** The predicted target miRNA and DEmiRNA intersected **(B)** The target mRNA and DEmRNA intersected.

Through the CircInteractome database, we predicted a total of 395 target miRNAs that bind to 15 DEcircRNAs. Following that, using the Venn diagram method, the predicted target miRNA and DEmiRNA were intersected, and 52 intersection miRNAs were obtained, as shown in Figure 3A. Next, genes identified in the TargetScan database were selected as potential target mRNAs. Then, the above-mentioned intersection miRNAs were predicted to obtain target 9,607 mRNAs. By intersecting the target mRNA and DEmRNA, 10 intersection mRNAs were obtained, as shown in Figure 3B.

### Construction of the regulatory network

**FIGURE 4:**
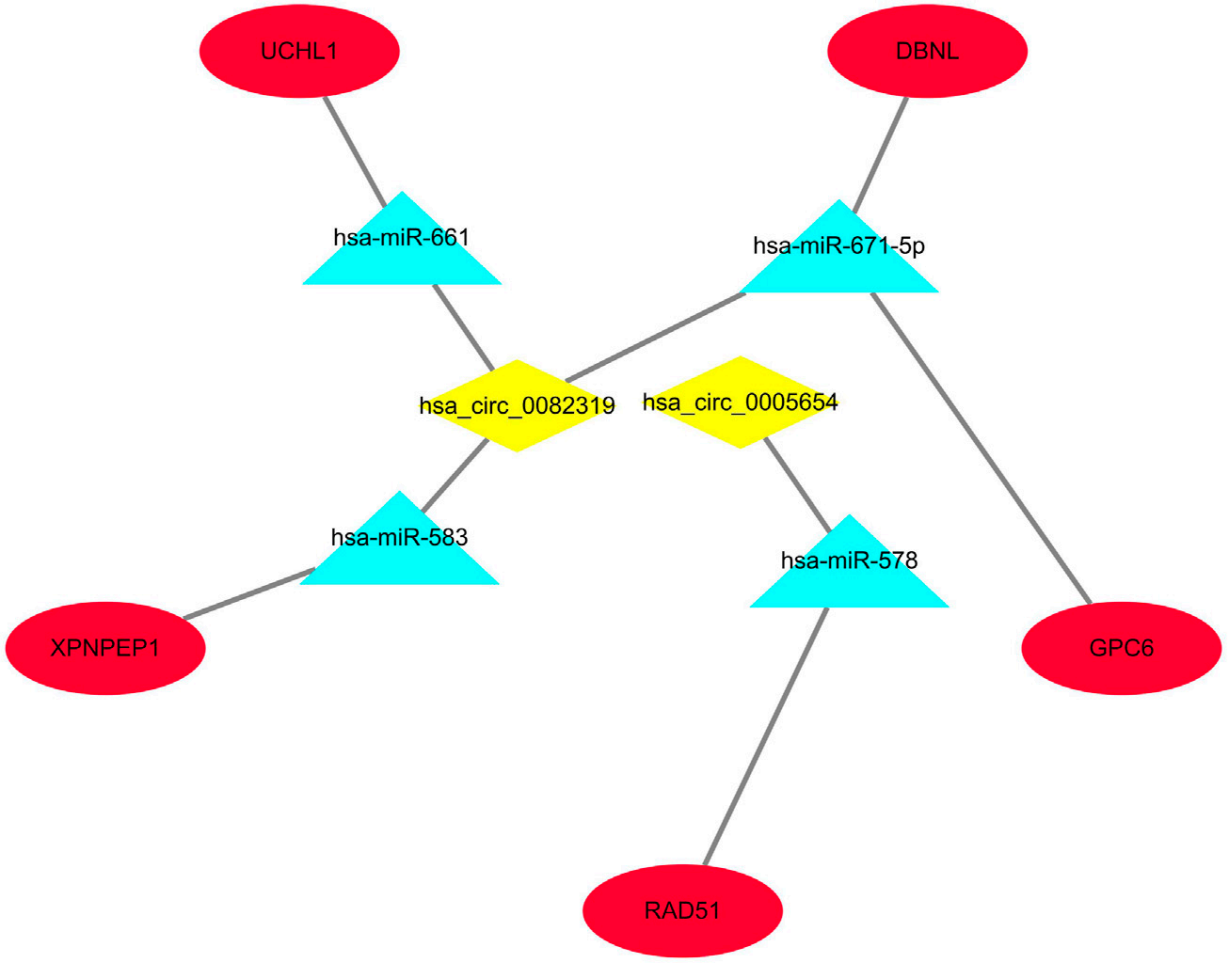
The visualized ceRNA regulatory network was constructed by Cytoscape software. The diamond represents 2 downregulated circRNAs, the triangle represents 4 upregulated miRNAs and the ellipse represents 5 downregulated mRNAs.

**FIGURE 5:**
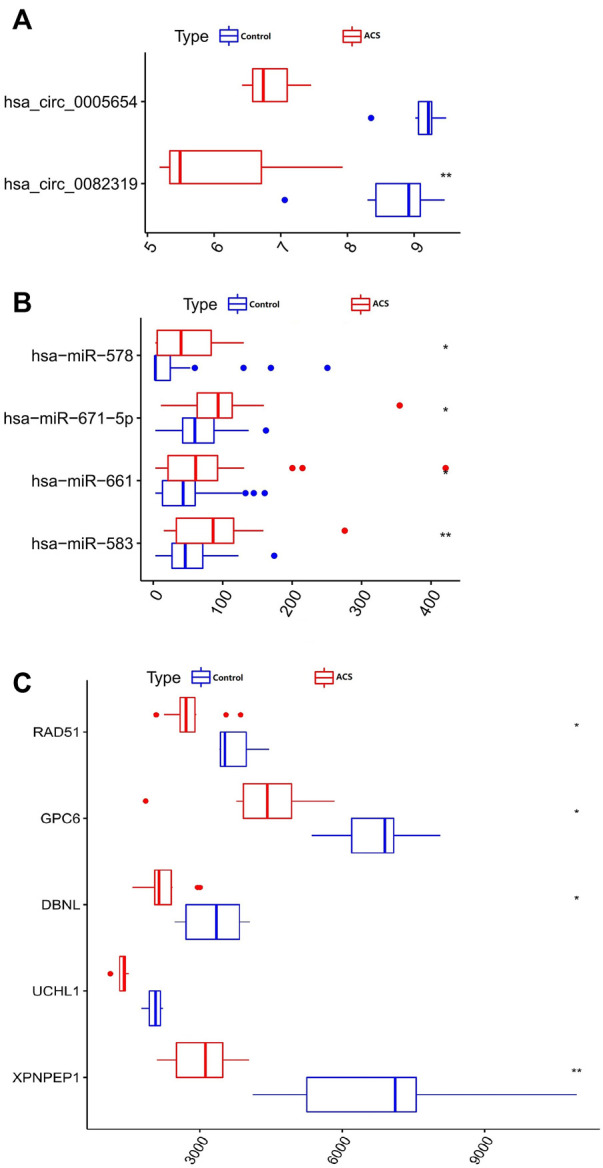
Boxplot of circRNA, miRNA, and mRNA in the ceRNA regulatory network. **(A)** 2 circRNAs. **(B)** 4 miRNAs. **(C)** 5 mRNAs. *p < 0.05, **p < 0.01.

Only genes that meet the following criteria will be selected for inclusion in the ceRNA network: 1) All genes must be differentially expressed; 2) circRNAs and mRNAs have a binding relationship with miRNAs at the same time; 3) RNAs (circRNA, mRNA) and miRNAs that meet the above binding relationship must be negatively regulated. Then, through Cytoscape software, a ceRNA regulatory network (circRNA-miRNA-mRNA) was constructed (Figure 4), including 2 circRNAs (hsa_circ_0082319, hsa_circ_0005654), 4 miRNAs (hsa-miR-583, hsa-miR-661, hsa-miR-671-5p, hsa-miR-578), and 5 mRNAs (*XPNPEP1, UCHL1, DBNL, GPC6,* and *RAD51*). Meanwhile, the “ggpubr” package and “reshape2” package in R were used to visualize the circRNA, miRNA, and mRNA in the ceRNA regulatory network (Figures 5A–C). The structure pattern diagrams and basic characteristics of the 2 circRNAs, are shown in Supplementary Table S1; Supplementary Figure S1.

### GO function annotation and KEGG pathway analysis

**FIGURE 6:**
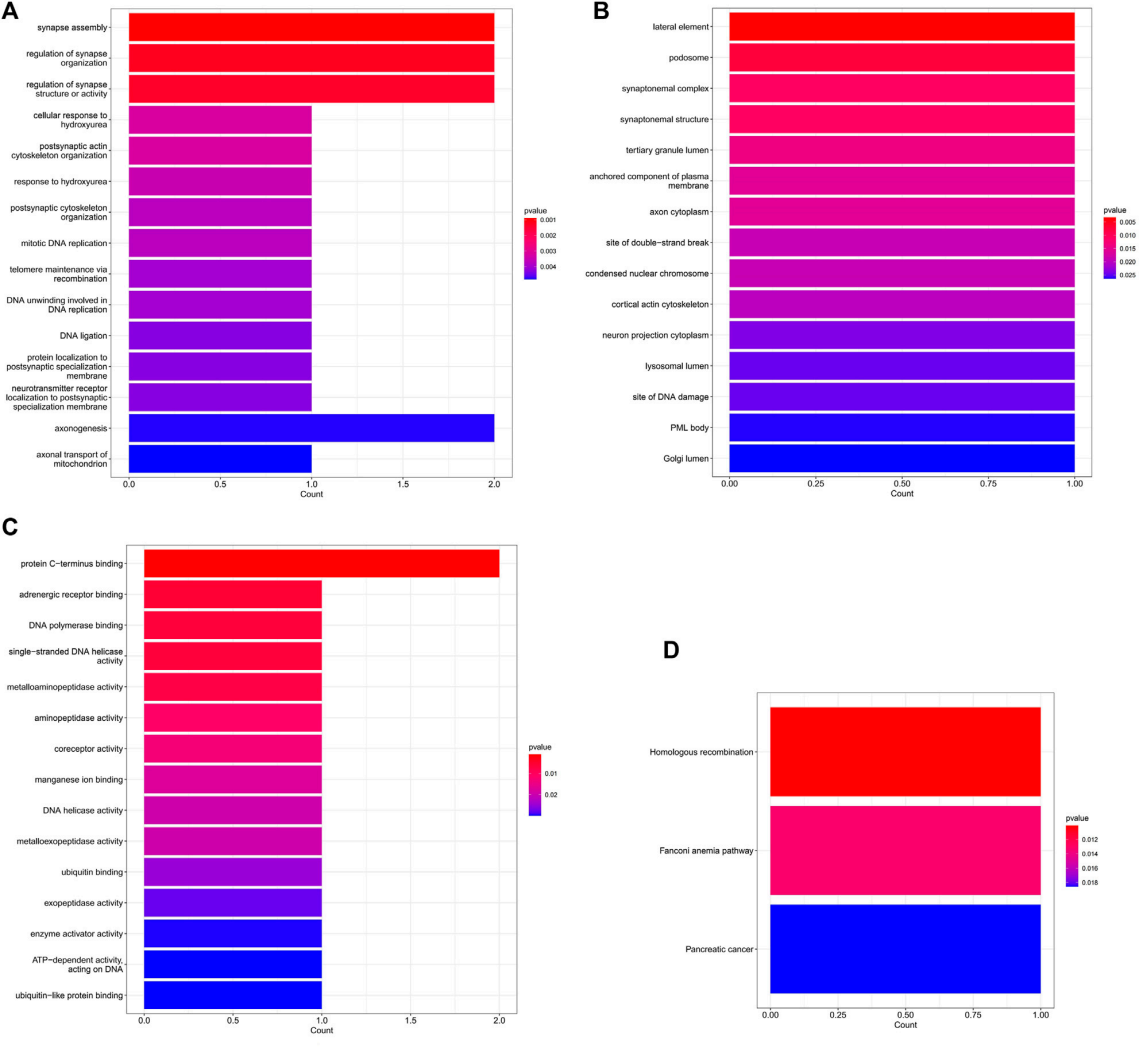
The GO and KEGG enrichment analysis of mRNA in the ceRNA network. **(A)** The top 15 GO terms of biological process. **(B)** The top 15 GO terms of cellular component. **(C)** The top 15 GO terms of molecular function. **(D)** The 3 KEGG pathways.

In the ceRNA regulatory network, GO analysis of 5 mRNAs indicated a major enrichment in: cortical actin cytoskeleton, protein C-terminus binding and adrenergic receptor binding. The histogram is displayed in Figures 6A–C. The results of the KEGG pathway analysis indicated that the main enrichment occurred in homologous recombination. (Figure 6D).

### Quantitative RT-PCR verification of hub genes

**FIGURE 7:**
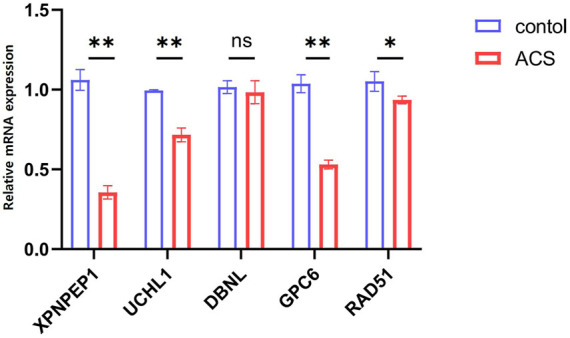
The expression levels of five hub genes in the ceRNA network analyzed by quantitative RT-PCR.*p < 0.05, **p < 0.01. Control group: *n* = 5, ACS group: *n* = 8.

The information of datasets and verification patient were shown in the Supplementary Table S2. In order to keep consistency with patients in the GEO datasets, our research objects included STEMI, NSTEMI, and UA patients, and the collection time of samples was limited to 12 h after the onset of symptoms. The expression of the five hub genes (*XPNPEP1, UCHL1, DBNL, GPC6,* and *RAD51*) in the ceRNA network was analyzed by quantitative RT-PCR. Between the control and ACS groups, we found that the *XPNPEP1, UCHL1, GPC6,* and *RAD51* genes had significantly decreased expression in ACS patients. The *DBNL* gene did not show any significant difference (Figure 7).

### Predicting the protective value of characteristic biomarkers in acute coronary syndrome

**FIGURE 8:**
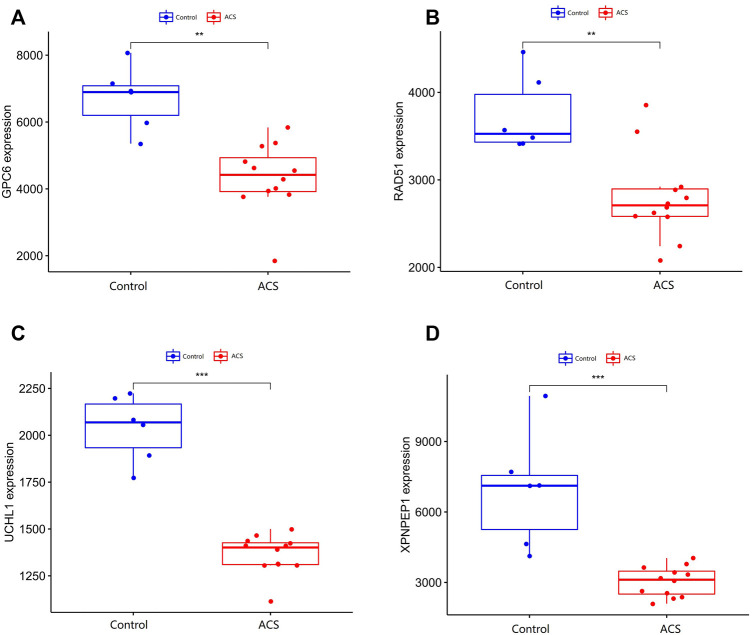
Validation of differentially expressed genes in ACS. **(A–D)** shows the expression of differentially expressed genes in the GSE95368 data set in ACS and non-ACS patients. The red box represents gene expression in the ACS group, and the bule box represents gene expression in the healthy control group.*p < 0.05, **p < 0.01, ***p < 0.01.

**FIGURE 9:**
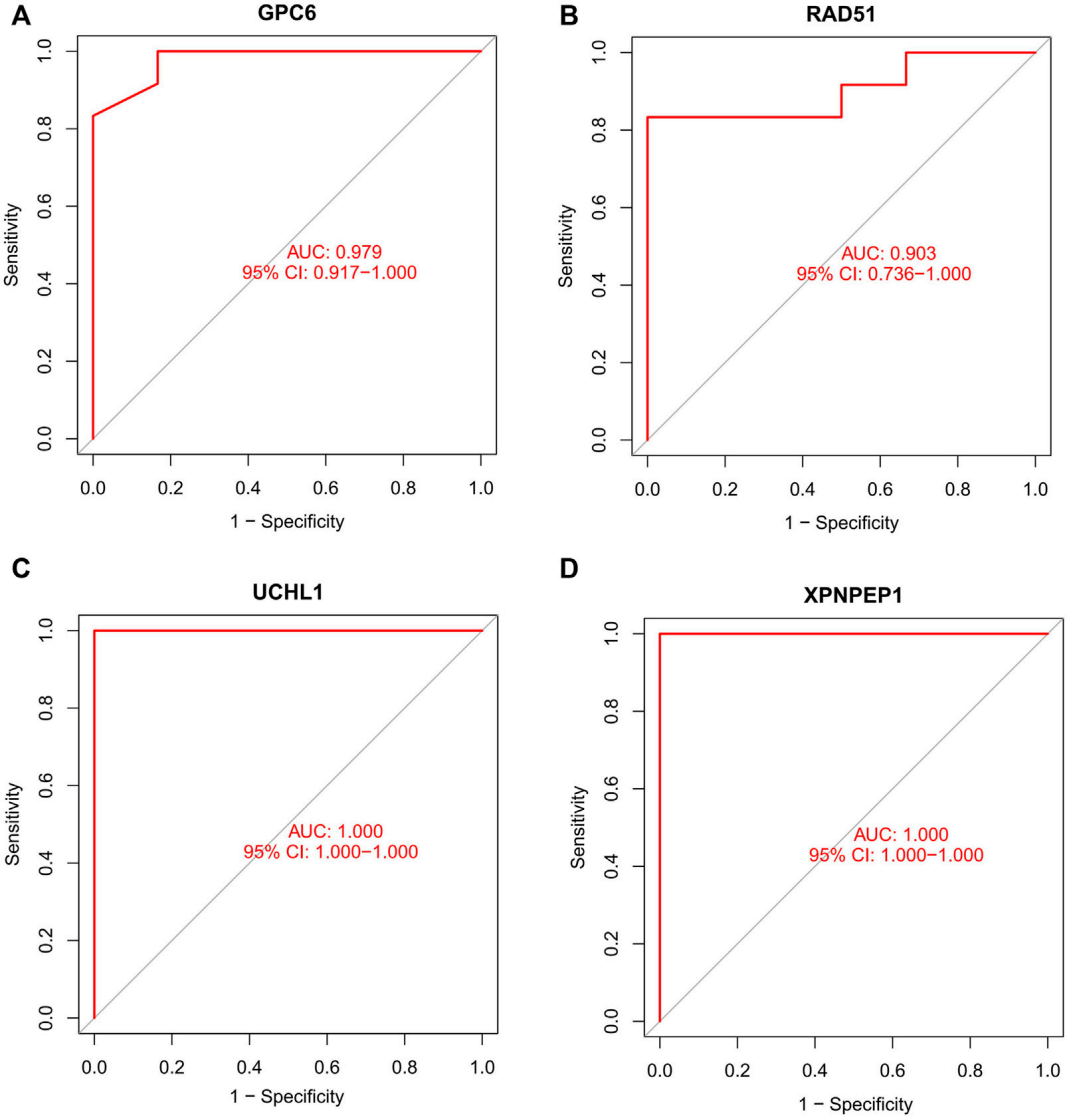
Receiver operating characteristic (ROC) curve of differentially gene’s ability to exclude ACS diagnosis in the GSE95368 dataset.

We compare the different expressions of *XPNPEP1, UCHL1, GPC6,* and *RAD51* in the GSE95368 and GSE60993 datasets (Figure 8; Supplementary Figure S2). Furthermore, we used the ROC curve to analyze the 4 mRNA from the GSE95368 and GSE60993 datasets for predicting and verifying characteristic biomarkers in ACS respectively. In the GSE95368 dataset, ROC curve analysis discovered that the *XPNPEP1, UCHL1, GPC6,* and *RAD51* genes have important clinical significance in preventing the occurrence and excluding diagnosis of ACS (Figures 9A–D). The AUC values of *XPNPEP1* in the GSE95368 dataset and the GSE60993 dataset were 1.000 (95% CI: 1.000–1.000, *p* < 0.05) and 0.777 (95% CI: 0.577–0.934, *p* < 0.05) dataset, respectively (Figure 9D; Supplementary Figure S3D). Logistic regression analysis showed that the *XPNPEP1* gene was significantly related to the occurrence of MACE in ACS patients [OR = 7.408 (95% CI: 0.762–72.009, *p* < 0.05)].

### Expression of XPNPEP1 in cardiomyocytes

**FIGURE 10:**
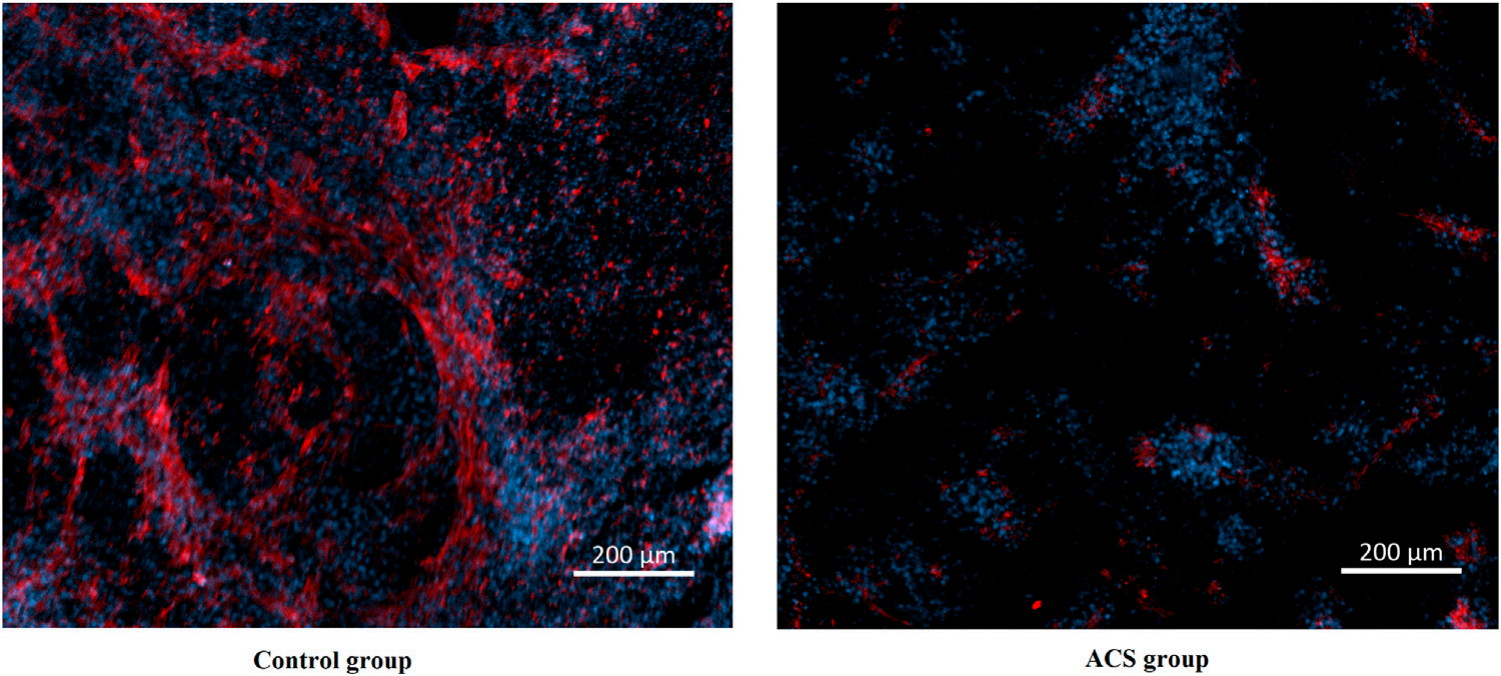
Immunofluorescence stain images of cardiomyocytes derived from human induced pluripotent stem cells. XPNPEP1: red. DNA: blue.

To explore the relationship between XPNPEP1 and ACS, we performed immunofluorescence staining on hiPSC-CM from patients with ACS and healthy human respectively. The results of immunofluorescence staining indicated that XPNPEP1 (red fluorescence) was expressed low in the cardiomyocytes of ACS patients (Figure 10).

### Correlation analysis between acute coronary syndrome hub genes and immune infiltrating cells

**FIGURE 11:**
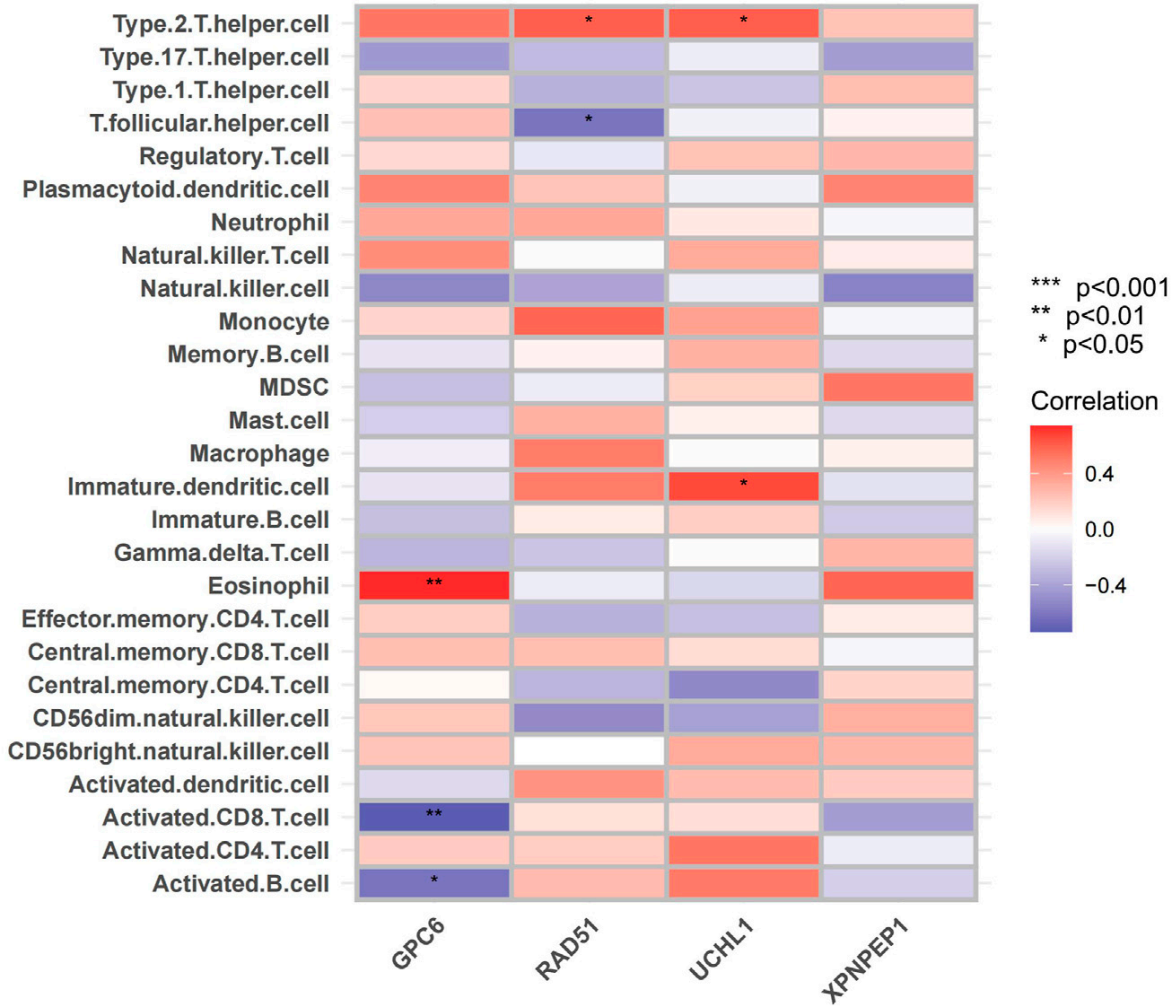
Immune cell infiltration analysis. The correlation analysis between immune cells: The red square represents positively correlated with upregulated immune cells. The bule square represents negatively correlated with decreased immune cells.

Considering that ACS has been shown to be infiltrated with a large number of immune cells in previous studies (Kyaw et al., 2021). We analyzed the infiltrating immune cells between the different groups’ mRNA in ACS patients (Supplementary Figures S4, S5). Moreover, we analyzed the relationship between the expression of the four hub genes and immune cell infiltration in ACS patients using the ssGSEA method (Figure 11). The results were as follows: *GPC6* was positively correlated with Eosinophil. RAD51 was negatively correlated with T follicular and positively correlated with Type 2 T helper cell. *UCHL1* was positively correlated with Imumature dendritic cell and Type 2 T helper cell. *XPNPEP1* levels were not statistically significantly correlated with immune cell infiltration.

## Discussion

In community, the incidence of missed ACS cases is 3.8 per 1,000 person-years among people aged over 55 years (de Torbal et al., 2006). Electrocardiogram and cardiac troponin I can provide effective help for rapid diagnosis, but their time window, specificity, and sensitivity have certain limitations (Lindahl, 2001; Chang et al., 2008). Therefore, it is necessary to study new biomarkers for early diagnosis or exclude ACS. The Gene chip microarray system has been widely employed in heart disease research since the development of gene chip technology (Chen et al., 2019). CircRNAs have long been thought to be a critical regulator in the pathogenesis of ACS, and their aberrant expression has been shown to have a major impact on disease progression (Cai et al., 2019; Si et al., 2020; Zhang et al., 2020).

CircRNA affects gene transcription and regulation by interacting with miRNA, mRNA, or protein *via* the ceRNA mechanism (Lasda and Parker, 2014). In our ceRNA networks, it was found that the downregulation of circRNA (hsa_circ_0082319 and hsa_circ_0005654) and downregulation of mRNA (*XPNPEP1, UCHL1, DBNL, GPC6,* and *RAD51*) formed a competitive relationship, which jointly led to the occurrence of ACS. It indicates that the expression of *UCHL1, DBNL, GPC6, RAD51,* and *XPNPEP1* can protect against ACS.

Every physician knows that the typical symptom of ACS is chest pain or pressure radiating across the chest and down the left arm, but some ACS can also present as dyspnoea, isolated jaw or arm pain, bilateral arm pain and back pain, or nausea and vomiting without any pain (Brieger et al., 2004; Wu et al., 2018; Chang et al., 2019; Kwok et al., 2021), which can lead both patients and physicians to fail to recognize ACS. Because of these factors, objective detection is critical for confirming or excluding ACS. Our study found that the *XPNPEP1* gene is the protective gene of ACS. By further plotting and analyzing the ROC curve, it can be utilized as a gene to exclude diagnosis. Outpatient follow-up of ACS patients found that the *XPNPEP1* gene was related to the occurrence of MACE in patients. Immunofluorescence staining showed that *XPNPEP1* was low expressed in the control group compared with ACS patients. The possible mechanism is that inhibition of *XPNPEP1* expression leads to the activation of CARD8 (caspase activation and recruitment domain 8) (Rao et al., 2022), which regulates the expression of cytokines and chemokines in endothelial cells and atherosclerotic lesions (Paramel et al., 2020). We speculate that the *XPNPEP1* gene indirectly affects the occurrence and development of atherosclerosis by participating in the function and metabolic mechanism of vascular endothelial cells. It is an important clue to clarify the regulatory mechanism of *XPNPEP1* at gene and protein levels in the pathogenesis of ACS.

It is known that *UCHL1* (Ubiquitin carboxyl-terminal hydrolase L1) is highly expressed and plays an important role in neurons, and it is usually used as a neuronal marker (Day and Thompson, 2010; Matuszczak et al., 2020). In recent years, there have also been some studies on the role of *UCHL1* in the cardiovascular system. Geng et al. (2022) compared the ischemic heart injury group with the control group and found the overexpression of *UCHL1* has a protective effect on myocardial injury after myocardial infarction. Upregulation of *UCHL1* can prevent cardiac remodeling and dysfunction after myocardial infarction by supporting autophagy flow and protein homeostasis (Wu et al., 2022). For the *GPC6* gene, a previous study found that compared to patients with heart failure after myocardial infarction, patients without heart failure had significantly lower glypican-6, suggesting that *GPC6* may be a protective gene for heart failure after myocardial infarction (Ozturk et al., 2021). Previous studies have found that RAD51 is a DNA damage repair molecule and it is involved in the pathogenesis of atherosclerosis (Davies et al., 2001; Singh et al., 2020). The conclusions of above studies are consistent with our results: *UCHL1, GPC6,* and *RAD*51 indirectly inhibit the occurrence of atherosclerosis or myocardial injury through various molecular biological level regulatory mechanisms.

ACS is not only an obstructive vascular disease, but also involves chronic vascular inflammation that causes atherosclerosis (Zhao and Mallat, 2019). For example, monocytes and macrophages play a pivotal role in the initiation, progression and instability of atherosclerotic plaque through multiple mechanisms such as necrosis and subsequent release of proinflammatory factors (Wei et al., 2018). It is not clear whether the hub genes in our study influence in the pathogenesis of ACS by immune cell infiltration. Therefore, we determined the relationship between the expression of three hub genes and immune cell infiltration by the ssGSEA method. The hub genes were highly enriched in immune-related or inflammation-related responses and pathways, which means that immune infiltration may be closely involved in the regulation of ACS pathogenesis.

The GO functional annotation revealed that hub mRNAs implicated in cortical actin cytoskeleton, protein C-terminus binding, and adrenergic receptor binding were overrepresented. Previous study has shown that the cortical actin cytoskeleton was involved in the pathogenesis of ACS through inflammatory and oxidative stress mechanisms (Hill et al., 2022). It is unknown how C-terminal protein plays a role in the pathophysiology of ACS. We speculate that the C-terminal protein may affect the pathogenesis of ACS as an inflammatory mediator (Hansson, 2005). According to a previous study, the adrenergic receptor blocker metoprolol has a special effect on neutrophils when inflammation intensifies, which provides cardiac protection (Clemente-Moragon et al., 2020). Adrenergic receptors are one of the targets for promoting the proliferation of adult cardiomyocytes and cardiac regeneration, which opens up a possible avenue for myocardial repair after myocardial infarction (Du et al., 2022). Through GO analysis of key mRNA, we found that the inflammatory mechanism is the basic pathological mechanism of ACS, and also explained that the adrenergic receptors are involved in the mechanism of myocardial damage repair after ACS.

The novelty of our study is that the results are based on the circRNA-associated ceRNA network in ACS patients. At the same time, the hub genes in the network were validated and analyzed by ROC curve and immune cells infiltration. At the same time, our research also has some deficiencies. On the one hand, because the sample size of the circRNA dataset is relatively small, no upregulated circRNA and mRNA were found in the ceRNA network of our study. On the other hand, detailed data for long-term follow-up to check for prognosis-predicting mRNAs is lacking. However, our research indirectly proves that the key RNAs may predict protective gene biomarkers of ACS and lays a solid framework for future research.

## Conclusion

Timely diagnosis and treatment of ACS can help to improve the prognosis of patients and reduce the economic and social damage. Our study constructed a circRNA-related ceRNA network in ACS. We found that hsa_circ_0082319 and hsa_circ_0005654 were involved in the regulation of *XPNPEP1, UCHL1, DBNL, GPC6,* and *RAD51* expression or activity through sponging hsa-miR-583, hsa-miR-661, hsa-miR-671-5p and hsa-miR-578. The *XPNPEP1* gene could be a protective gene biomarker for ACS. Three other hub genes (*UCHL1, GPC6,* and *RAD51*) were significantly correlated with immune cells (Eosinophil, T follicular, Type 2 T helper cell and Imumature dendritic cell) in ACS.

## Data Availability

The datasets presented in this study can be found in online repositories. The names of the repository/repositories and accession number(s) can be found in the article/Supplementary Material.
