# Supplementary material for: Predicting protective gene biomarker of acute coronary syndrome by the circRNA-associated competitive endogenous RNA regulatory network
Source: Front Genet. 2022 Oct 19;13:1030510. doi: 10.3389/fgene.2022.1030510 (PMC9627163; doi:10.3389/fgene.2022.1030510)
Supplement: Supplementary file 1 [file DataSheet1.docx]

***Supplementary Material***

**Supplementary table S1**. List of the primer sequences used for RT-PCR

| mRNA Name | Description | Sequence (5′ to 3′) |
| --- | --- | --- |
| GAPDH | Forward  Reverse | GGTCTCCTCTGACTTCAACA  AGCCAAATTCGTTGTCATAC |
| XPNPEP1 | Forward  Reverse | CTTGATCATTCCTACAGGCATCT  CAAGGCAGTGACCACAAACC |
| UCHL1 | Forward  Reverse | AAGGCCAATGTCGGGTAGATG  GACTTCTCCTTGCTCACGCT |
| DBNL | Forward  Reverse | TCCTCATCAACTGGACAGGC  ATGCACTCAGGCTCCACATC |
| GPC6 | Forward  Reverse | GGATCGGGGCTGTGATTCTT  TCTTAAGTGTTCCCCTGCGAT |
| RAD51 | Forward  Reverse | GAGACCGAGCCCTAAGGAGA  TCTGCATTGCCATTAGCTCCA |

**Supplementary table S2.** The fundamental properties of the two differentially expressed circRNAs that involved in ceRNA network.

| CircRNA ID | Strand | Position | length | Fold change | Gene symbol |
| --- | --- | --- | --- | --- | --- |
| hsa_circ_0082319 | - | chr7:129662158-129679387 | 17229 | 1031 | ZC3HC1 |
| hsa_circ_0005654 | - | chr4:121675707-121732604 | 56897 | 758 | PRDM5 |

**Supplementary table S3.** Basic data from the Gene Expression Omnibus (GEO) database, as well as verification patients

| Dataset ID | RNA type | Array platform | Sample size | Sample acquisition time | Tissue type |
| --- | --- | --- | --- | --- | --- |
| GSE197137 | circRNA | GPL21825 | Control:6  STEMI:3 | within 12 hours after symptom | Peripheral blood |
| GSE31568 | miRNA | GPL9040 | Control:70  AMI:20 | within 10 hours after symptom | Peripheral blood |
| GSE95368 | mRNA | GPL23119 | Control:6  AMI:12 | within 24 hours of diagnosis | Peripheral blood |
| GSE60993 | mRNA | GPL6884 | Control: 7  STEMI: 7  NSTEMI: 10  UA: 9 | within 4 hours after symptom | Peripheral blood |
| Verification patients | / | / | Control: 5  STEMI: 3  NSTEMI: 2  UA: 3 | within 12 hours after symptom | Peripheral blood |

STEMI: ST-elevation myocardial infarction.

NSTEMI: Non-ST-elevation myocardial infarction.

UA: Unstable angina.


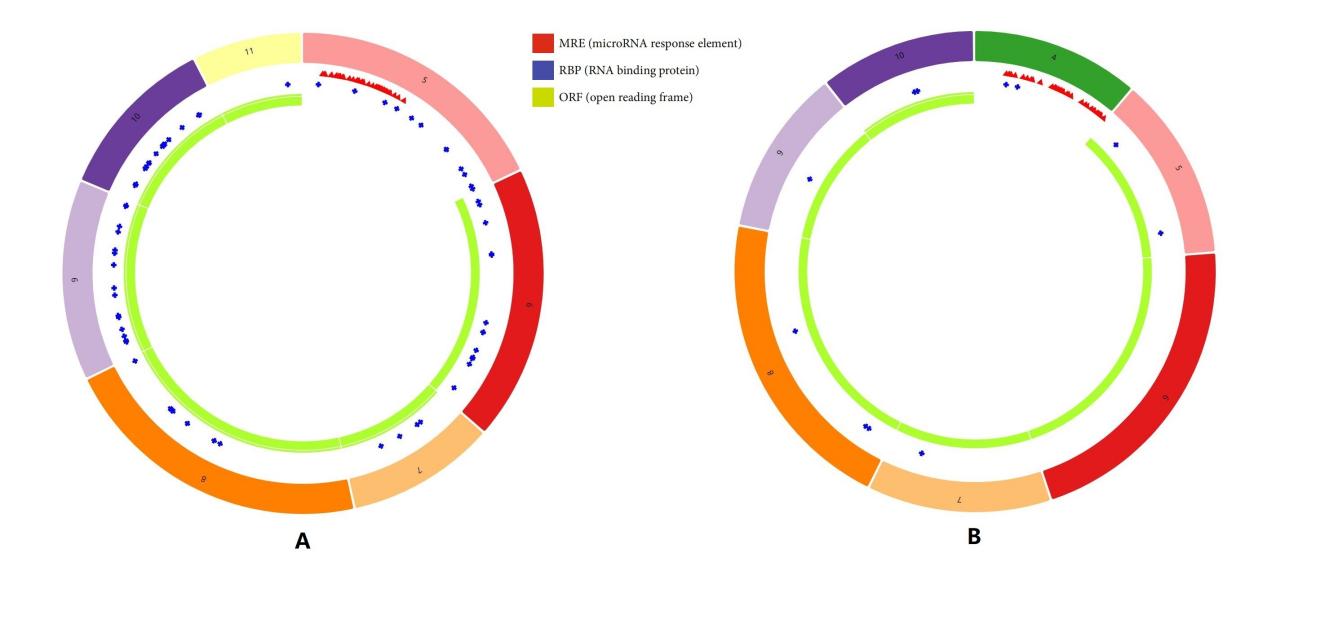


**Supplementary figure S1**. Structural patterns of circRNAs

The fluorescent green part represents the protein that circRNA may encode, namely Open Reading Frame (ORF). The blue part represents the location of circRNA bound to the protein (RBP), and the position of the small red triangle represents the binding position of the circRNA to the miRNA (MRE).


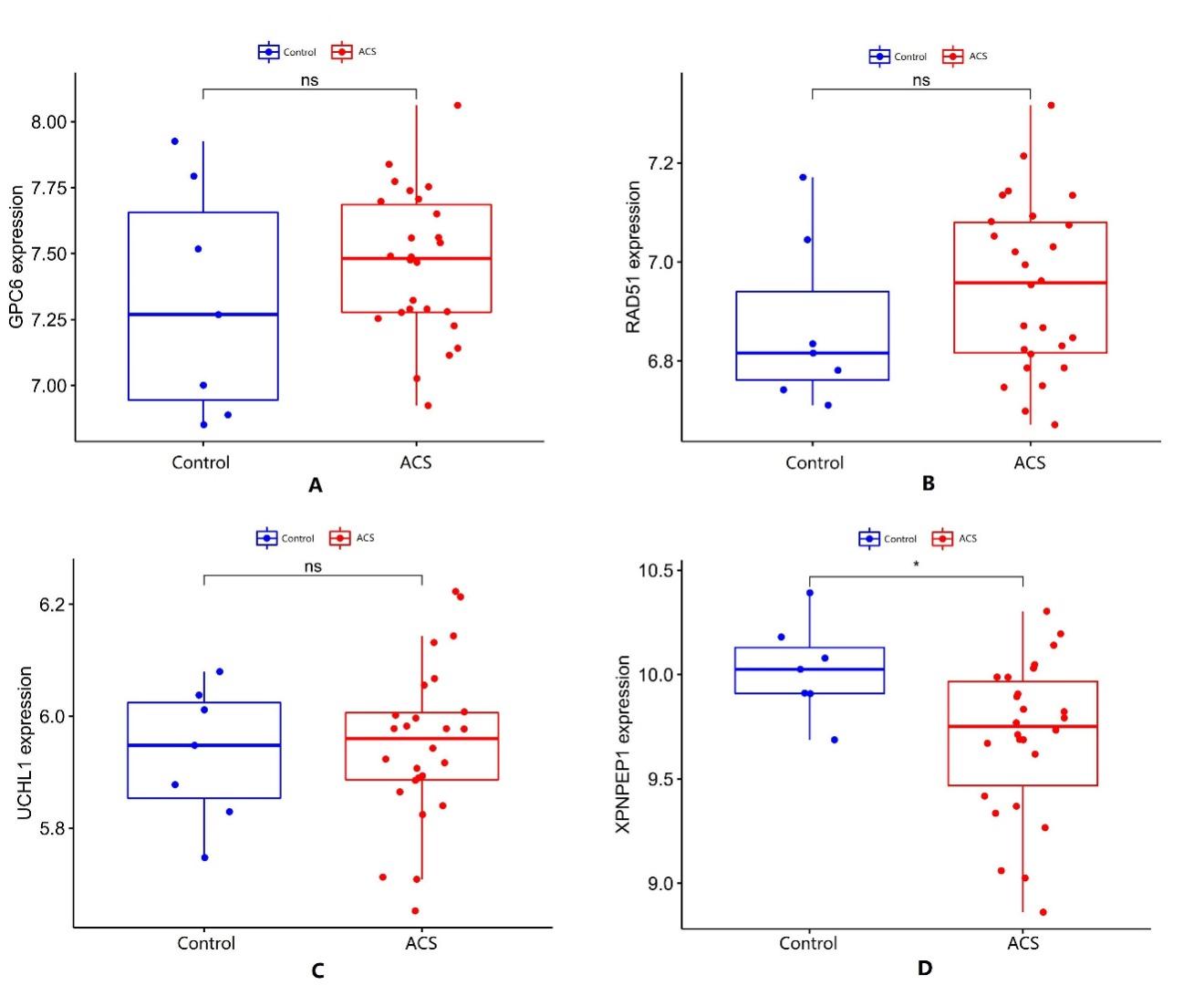


**Supplementary figure S2**. Validation of differentially expressed genes in ACS. The figure (A–D) shows the expression of differentially expressed genes in the GSE60993 data set in ACS and non-ACS patients. The red box represents gene expression in the ACS group, and the bule box represents gene expression in the healthy control group.


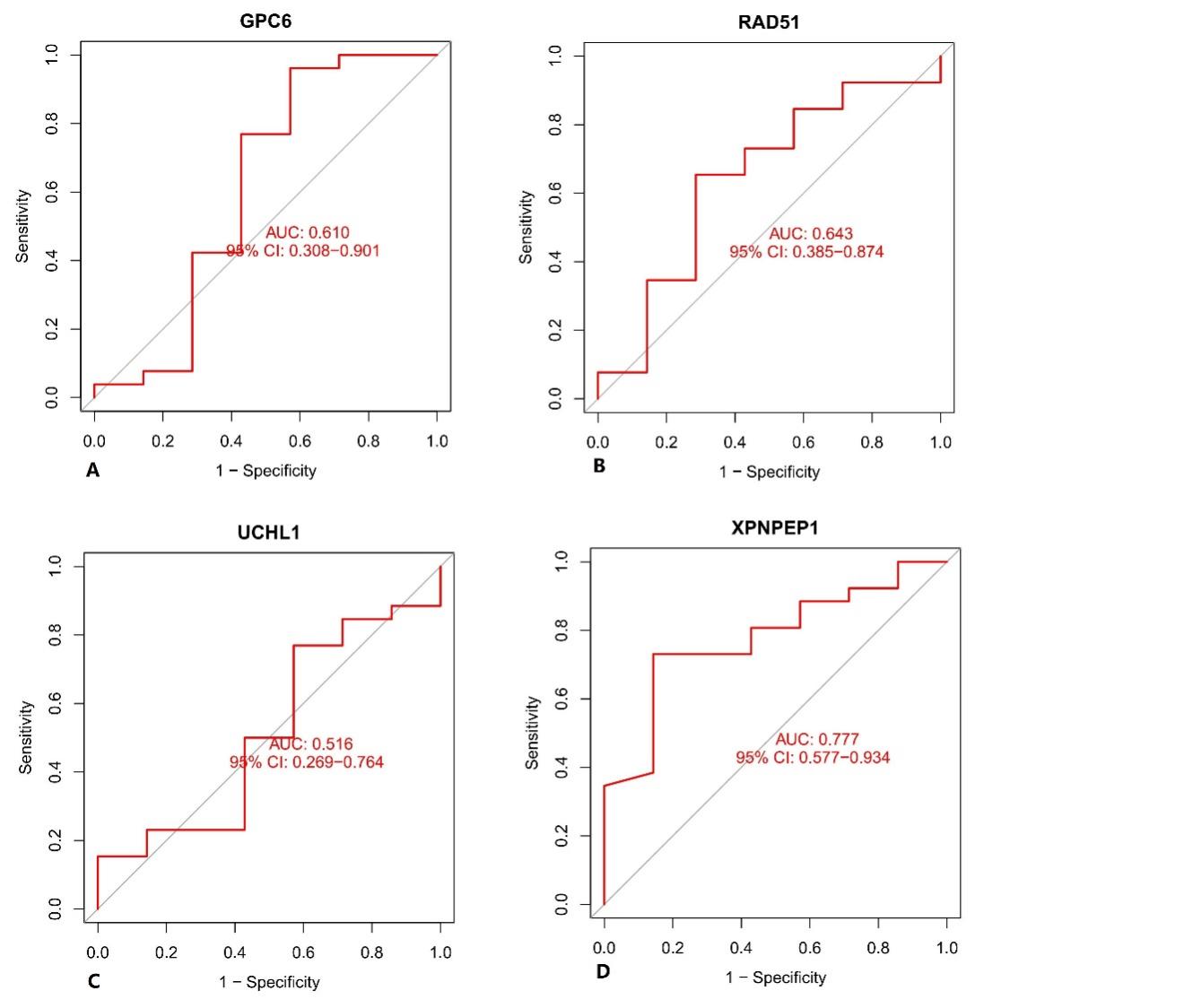


**Supplementary figure S3.** Receiver operating characteristic (ROC) curve of differentially gene’s ability to exclude ACS diagnosis in the GSE60993 dataset.


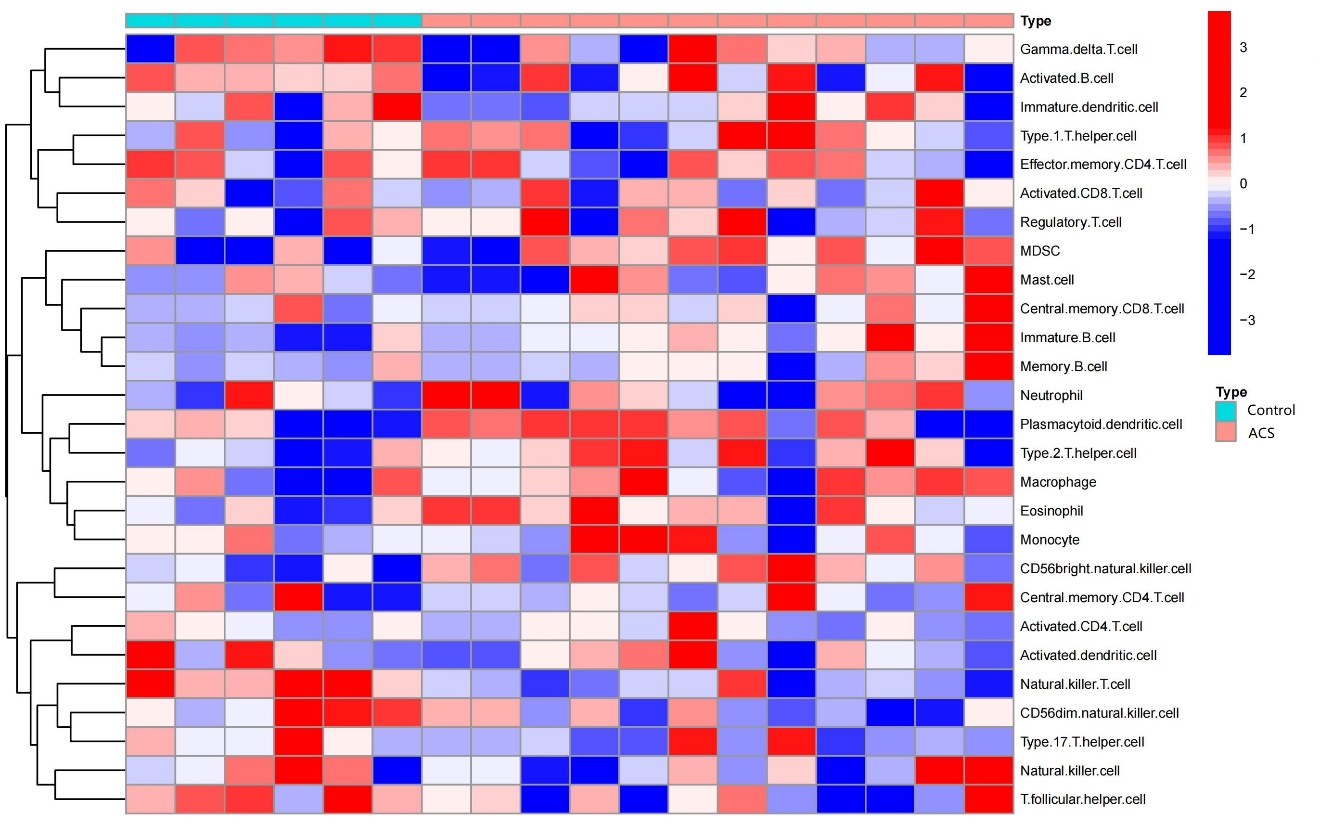


**Supplementary figure S4.** Heatmap illustrated the correlation between ACS patients and the immune cells.


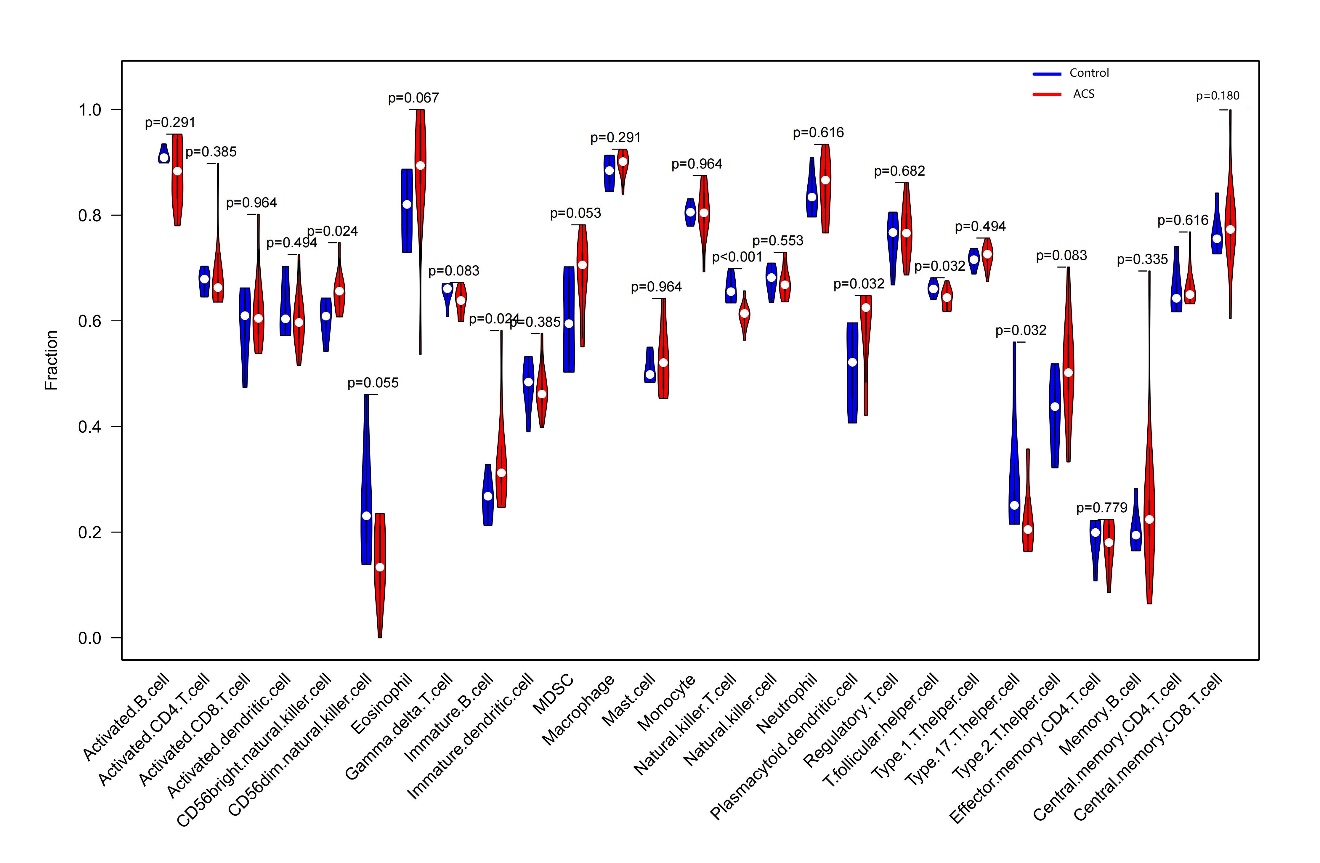


**Supplementary figure S5.** Violin plot showing the significant changes of the immune infiltration level in the patients with ACS compared to the control group.
